# Assessing the impact of Indiana legislation on opioid-based doctor shopping among Medicaid-enrolled pregnant women: a regression analysis

**DOI:** 10.1186/s13011-021-00366-x

**Published:** 2021-04-06

**Authors:** Sukhada S. Joshi, Nicole Adams, Yuehwern Yih, Paul M. Griffin

**Affiliations:** 1 School of Industrial Engineering, Purdue University, West Lafayette, IN 47906 USA; 2 School of Nursing, Purdue University, West Lafayette, IN 47906 USA; 3 Department of Industrial and Manufacturing Engineering, Penn State University, 310 Leonhard Building, University Park, PA 16801 USA

**Keywords:** Opioid prescriptions, Doctor shopping, Legislation, Medicaid, Pregnancy

## Abstract

**Background:**

States have passed various legislative acts in an attempt to reduce opioid prescribing and corresponding doctor shopping, including prescription drug monitoring programs. This study seeks to determine the association between two state-based interventions enacted in Indiana and the level of doctor shopping among Medicaid-enrolled pregnant women.

**Methods:**

Indiana Medicaid claims data over the period of January 2014 to March 2019 were used in a regression model to determine the longitudinal change in percentage of pregnant women engaged in doctor shopping based on passage of Indiana Administrative Code Title 884 in 2014 and Public Law 194 in 2018. The primary reasons for prescribing were also identified.

**Results:**

There were 37,451 women that had both pregnancy and prescription opioid claims over the time horizon. Of these, 2130 women met the criteria for doctor shopping. Doctor shopping continued to increase over the time between the passage of the two interventions but decreased after passage of Public Law 194.

**Conclusion:**

The decrease in doctor shopping among Medicaid-enrolled pregnant women after passage of Public Law 194 points to the importance of addressing this issue across a broad set of healthcare professionals including nurse practitioners and physician assistants. It is also possible that the potential punitive component in the Law for non-compliance played a role.

## Introduction

Over 42% of opioid-related overdose deaths for US women were due to prescription opioids in 2018 [1]. Prescription opioid use among pregnant women has risen significantly since 2000 [2, 3]. In 2019, 6.6% of pregnant women in the US reported prescription opioid use [4], though previous studies have found that 14 to 22% of Medicaid-enrolled pregnant women filled at least one opioid prescription during pregnancy [5]. In addition to harms to the mother, opioid use during pregnancy has also been associated with poor infant outcomes including neonatal abstinence syndrome (NAS). NAS incidence increased nationally from 1.6 per 1000 in-hospital births in 2004 to 8.8 per 1000 births in 2016 [6] with Medicaid covering 82% of those births in 2014 [7].

Opioid doctor shopping is the practice of using multiple concurrent prescribers to obtain opioid-based prescription drugs and may be due to reasons of misuse from addiction or diversion [8, 9]. Patients that doctor shop for opioids are at a greater risk of opioid use, injury, and overdose death [8, 10]. Studies on longitudinal trends of doctor shopping prevalence have been mixed. For example, one study found both an increase in morphine equivalent dose (MED) and doctor shopping in California from 2008 to 2012 [11], while another found a geographically widespread decline nationally over that same time period [12]. However, in many states, guidelines and recommendations have been implemented in an effort to reduce opioid prescribing for a variety of conditions [13, 14]. These include national guidelines such as those developed by the Centers for Disease Control and Prevention [15] and Veteran’s Administration [16] and numerous state level interventions [17].

There have been a variety of programs and/or legislation at the state level that attempt to reduce opioid prescribing, diversion, and misuse. For example, prescription drug monitoring programs (PDMP) in the form of electronic databases to track controlled substance prescriptions have been implemented in all 50 states. With the exception of a reduction in prescription opioid-related death rates [18, 19], evidence of their efficacy has been weak [20–23]. Patients that doctor shop for opioids generally receive prescriptions from high volume prescribers [24, 25] and do not receive a significant supply from episodic providers, thus potentially negating the effectiveness of prescription reduction efforts at episodic settings [25].

Indiana enacted two state-based interventions, Opioid Prescribing Requirements created by the Medical Licensing Board and filed under Indiana Administrative Code 884 IAC 5–6 in December 2013 and Indiana Public Law 194 in March 2018, the first states that prescribers “shall” consult the PDMP and the latter “requires” providers to do so prior to prescribing an opioid. The Opioid Prescribing Requirements targeted long-term prescribing (over 3 months) for doses over 15 MED per day or 60 pills per month. The new law passed in 2018 applied to single prescriptions and emergency departments. We use a regression discontinuity model on Medicaid claims to determine if there was an associated change in the rate of opioid-based doctor shopping among Medicaid-enrolled pregnant women following the introduction of these policies. We also use a page-rank algorithm to determine the most likely associated diagnosis claims that led to a prescription.

## Materials and methods

### Indiana opioid-based legislation

The state of Indiana implemented the PDMP program (INSPECT) in 2004 through the expansion of previous legislation [26]. In 2013, the Indiana Medical Licensing Board enacted emergency prescribing rules, which became permanent in 2014 as the Indiana Administrative Code 844 IAC 5–6. This prescribing standard for long-term opioid users outlines many components of assessment and care prior to beginning an opioid treatment plan which included advising prescribers to review the patients’ drug prescription history in INSPECT, schedule periodic visits for patients prescribed opioids, and obtain a signed patient agreement [27]. In March 2018, Senate Bill 221 passed and became Indiana Public Law 194 which required that beginning in January 2019, prescribers were required to review INSPECT prior to the prescribing of any opioids for any duration [28]. This legislation directly covers all medical practitioners and includes potential medical negligence penalties for non-compliance.

### Data

We analyzed Indiana Medicaid claims over the period of January 2014 to March 2019. The ICD9 and ICD10 codes (Table 1) were used to identify pregnant women that received an opioid prescription during pregnancy. Claims were matched to the national drug code directory [29] for prescription opiates. The billing national provider identifiers (NPI) were used to uniquely identify prescribers. Doctor shoppers were identified as pregnant women with at least one pair of consecutive claims less than 30 days apart prescribed by different providers. In order to estimate if the change in doctor shopping was due to a drop in the practice or due to organization change, we compared the frequency of doctor shopping incidents for each pregnant woman that had at least incident both pre- and post-passage of Public Law 194 (Q1 of 2018).

**Table 1:** ICD9/10 codes for pregnancy diagnosis

Diagnosis Code	ICD 9/10	Description
V22, V23	ICD 9	Pregnancy (Normal/High Risk)
O00.00-O9A.53	ICD 10	Pregnancy, childbirth and puerperium
Z33.1-Z33.3, Z34.00-Z34.93	ICD 10	Encounter for pregnancy

Claims that were characterized as part of an individual’s doctor shopping were dated by quarter (Q1 to Q4) each year on the basis of the claim date. The quarter that a pregnant recipient procured the most prescriptions is defined as her primary doctor shopping quarter. For each quarter we computed percent doctor shopping as 100*(number of pregnant women classified as doctor shoppers/number of pregnant women that received an opioid prescription during pregnancy).

### Statistical methods

We used a regression model across the time horizon, starting at the time of release of the Indiana Prescribing Guidelines (IAC 884 5–6, Q1 of 2014), to determine if there was a change in the rate of doctor shopping before and after the passage of Public Law 194 (Q1 of 2018). Quarters were used as the time interval since it was the smallest interval were there were a sufficient number of pregnant that met the criteria for doctor shopping in each period. Our time horizon consisted of 21 quarters. The regression model used is:

$$ \mathrm{Y}={\mathrm{a}}_0+{\mathrm{a}}_1\mathrm{I}+{\mathrm{a}}_2\mathrm{R}+{\mathrm{a}}_3\mathrm{I}\ast \mathrm{R} $$

where: I equal 1 if observation is on or after the passage of Public Law 194 (Q1 2018) and 0 otherwise; R is the time period (quarter) minus quarter of the intervention (so that the intervention is scaled to 0); and Y is the percent doctor shopping. The key parameters of interest are a_2_, which corresponds to the change in the percent doctor shopping after release of IAC 884 5–6, and the interaction coefficient a_3_, which corresponds to the difference in slopes before and after passage of Public Law 194.

We chose our regression method over other approaches such as interrupted time series (ITS) analysis [30] for multiple reasons. First, it is recommended that there be at least eight post intervention observations for ITS to be effective [31]. As our observations were quarterly, there were fewer than eight measurements after passage of Public Law 194. Second, our key parameters of interest were the slopes before and after the passage of this intervention. Our regression model directly estimates these parameters. Finally, visual inspection of the change in longitudinal change in doctor shopping percentage appears to be linear (Results), and hence a linear model seemed the most appropriate, which did not require the nonlinear flexibility that is possible through ITS. Note, however, that the assumptions of linear regression include that the residuals are independent and normally distributed. Proportions data, however, are bounded to the interval [0,1], and a method such as beta regression [32] could be a more appropriate model since a regression model may include predictions outside of this interval. However, we are again limited by the number of observations. We therefore consider the linear regression assumptions using the Durban Watson statistic to test for independence, residual plot to test for homoscedasticity, and the normal probability plot to test for normality.

## Results

From the Indiana Medicaid data, 37,451 women had both pregnancy and prescription opioid claims from Q1 2014 to Q1 2019. Of these, 2130 women met the criteria for doctor shopping. For these women, the diagnoses associated with claims of prescription opioids were primarily for abdominal pain including (in rank order): unspecified abdominal pain (ICD10 R109), right lower quadrant pain (ICD10 R1031), right upper quadrant pain (ICD10 R1011), pelvic and perineal pain (ICD10 R012) abdominal pain (ICD9 78,909 and 78,900), epigastric pain (ICD10 R103), lower left quadrant pain (ICD10 R1032), and lower abdominal pain unspecified (ICD10 R1030). The diagnoses percentages are shown in Table 2.

**Table 2:** ICD9/10 codes for diagnoses associated with claims of prescription opioids for the 2130 Medicaid-enrolled pregnant women

Diagnosis Code	ICD 9/10	Description	Number of Pregnant Women Classified as Not a Doctor Shopper (a)	Number of Pregnant Women Classified as Doctor Shopper (b)	Percent of Pregnant Women with Specified Diagnosis Code that were Classified as Doctor Shopper (b)/((a) + (b))	Percent of Doctor Shopping Pregnant Women with Diagnosis Code Out of Total (b)/2130
R109	ICD 9	Unspecified abdominal pain	702	341	32.7%	16.0%
R1011	ICD 9	Right upper quadrant pain	279	149	34.8%	7.0%
R102	ICD 9	Pelvic and perineal pain	264	147	35.8%	6.9%
R1031	ICD 9	Right lower quadrant pain	272	131	32.5%	6.2%
78,900	ICD 10	Abdominal pain, unspecified site	263	108	29.1%	5.1%
R1013	ICD 9	Lower abdominal pain, unspecified	257	107	29.4%	5.0%
78,909	ICD 10	Abdominal pain, other specified site	274	106	27.9%	4.9%
R1030	ICD 9	Lower abdominal pain, unspecified	230	99	30.1%	4.7%
R1032	ICD 9	Left lower quadrant pain	198	91	31.5%	4.3%

**Fig. 1:**
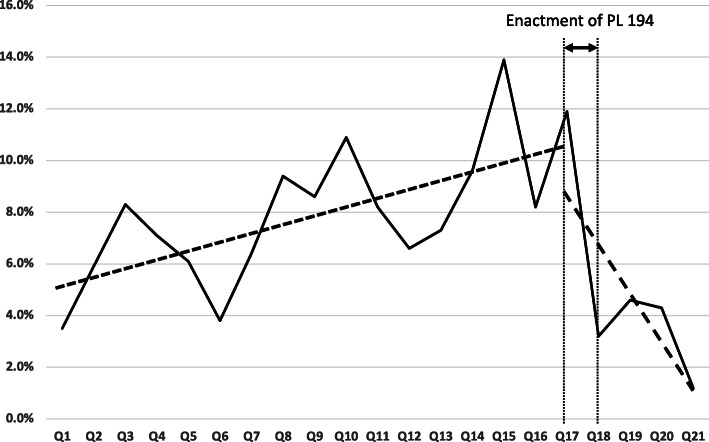
Percent of pregnant women with an opioid prescription that doctor shop over the planning horizon. Q1 2018 was the quarter where Public Law 194 [28] was passed and Q2 2018 the quarter that it was enacted

Figure 1 shows the change in percent drug shopping among Medicaid-enrolled pregnant women over the time horizon. The regression results for the coefficients are shown in Table 3. The adjusted R^2^ was 0.475 and the F statistic for the analysis of variance was significant (*p* = 0.003). The predicted value and observed values with confidence interevals are shown in Table 4.

**Table 3:** Results of regression model (*n* = 21) for percentage of pregnant women doctor shopping over time, where I is an indicator value that equals 1 if observation is on or after when Public Law 194 [28] was passed and 0 otherwise, R is the time period minus the time when Public Law 194 was passed, and I*R is the interaction of these two independent variables

Variable	Coefficient Value	Standard Error	***p***-value
Intercept	0.106	0.012	< 0.000
I	−0.015	0.021	0.493
R	0.003	0.001	0.014
I*R	−0.024	0.007	0.004

**Table 4:** Observations, confidence intervals, and regression predicted values for the 21 quarters

Quarter	Proportion of Pregnant Woment that Met Conditions of Doctor Shopping	95% Confidence Interval	Predicted Value from Regression
1	0.035	(0.019, 0.050)	0.049
2	0.059	(0.043, 0.756)	0.053
3	0.083	(0.061, 0.104)	0.057
4	0.071	(0.054, 0.088)	0.061
5	0.061	(0.046, 0.075)	0.065
6	0.038	(0.029, 0.048)	0.069
7	0.064	(0.049, 0.078)	0.073
8	0.094	(0.076, 0.112)	0.077
9	0.086	(0.070, 0.102)	0.081
10	0.109	(0.091, 0.127)	0.085
11	0.082	(0.068, 0.096)	0.089
12	0.066	(0.053, 0.079)	0.093
13	0.073	(0.056, 0.089)	0.097
14	0.096	(0.079, 0.112)	0.101
15	0.139	(0.118, 0.161)	0.105
16	0.082	(0.070, 0.093)	0.096
17	0.119	(0.103, 0.135)	0.079
18	0.032	(0.026, 0.038)	0.064
19	0.046	(0.039, 0.054)	0.048
20	0.043	(0.038, 0.049)	0.031
21	0.012	(0.009, 0.014)	0.015

Both the time period R and interaction term I*R were significant. The positive value of a_2_ implies that the percentage of pregnant women that engaged in doctor shopping increased from the time of release of Title 884 to the passage of Public Law 194 by an absolute rate of 0.3% per quarter. The negative value of a_3_ implies that the percent of pregnant women engaging in doctor shopping decreased after passage of Public Law 194 by an absolute rate of 2.4% per quarter. The value of a_0_ implies that the overall average of percentage of pregnant women engaging in doctor shopping over the entire horizon was 10.6%.

With regards to the linear regression assumptions, none of the predicted values fell on or below 0. In addition, the Durban Watson statistic for the regression was 2.23, which exceeds the critical value of 1.83 [33] and implies the regression errors do not have positive autocorrelation (i.e., are independent). The residuals plot (data not shown) appears to be homoscedastic, and the normal probability plot (data not shown) is close to linear.

**Fig. 2:**
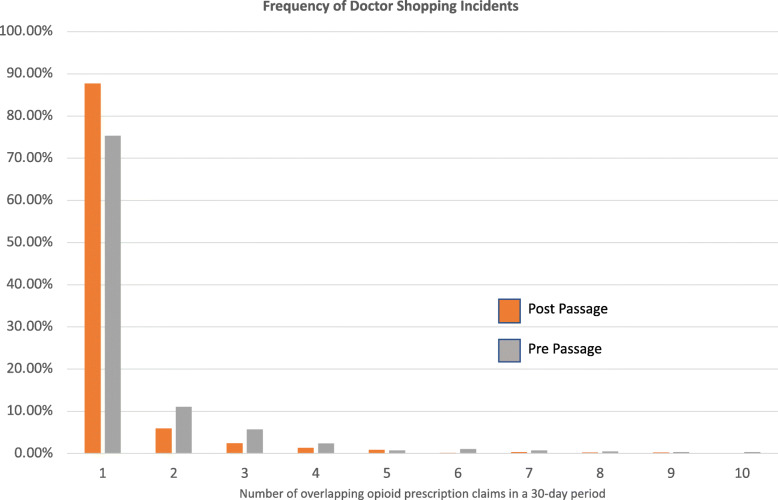
Percent of women that met the definition of doctor shopping by number of overlapping opioid prescription claims in a 30-day period

For pregnant women that doctor shopped, the percentage of times that they had a single overlapping opioid prescription claim (i.e., one incident) was 75.3% prior to passage of Public Law 194 and 87.7% after passage. However, whenever the number of incidents was greater than one (from two to 31 overlapping claims), the percentage was lower post-passage compared to pre-passage. The only exception was for five incidents (0.75% post-passage compared to 0.85% pre-passage). Figure 2 shows the comparison.

## Discussion

Although MEDs for all opioids per day decreased after introduction of the Opioid Prescribing Requirements from the Medical Licensing Board [34], our results show that the practice of doctor shopping among Medicaid-enrolled pregnant women significantly increased. The implication is that when supply became limited, individuals with an opioid use disorder sought alternatives [35, 36]. Doctor shopping among this group increased until the passage of Public Law 194, when a significant decrease occurred. Although Public Law 194 did not take effect until the first quarter of 2019, it appears that many prescribers adjusted their practices once the law was passed. This may explain the steep drop in doctor shopping seen in the first quarter of 2018 as prescribers prepared their systems and altered their practices to be ready when the law took effect. There is another significant drop in Q1 of 2019, which coincides with the formal enactment of the new law. However, because it is a single data point it is difficult to make definitive conclusions about the effect of the law and it is unknown if this drop was sustained over time.

The decreases seen in doctor shopping behavior are a direct reflection of the prescribing practices among Indiana providers. This points to the importance of legislation that addressed single prescriptions and not just long-term opioid prescribing. It is also possible that the potential punitive component in the Law for non-compliance played a role. In addition, the more complex and prescriptive nature of Public Law 194 required many steps by physicians for compliance. This may have led to effective practice changes when compared to the singular act of checking the PMDP as defined in the Opioid Prescribing Requirements from the Medical Licensing Board and being given the autonomy to use that information how the physician saw fit. Note that from Fig. 2, all frequencies higher than one incident of doctor shopping for the population had lower percentages after passage of Public Law 194 compared to prior and supports the case that was is being observed is actually due to the legislation and not just due to some organizational change.

Pain often occurs as a symptom of other medical conditions and diagnoses. It is interesting to note that a significant number of diagnoses for opioid prescriptions provided to Medicaid-enrolled pregnant women categorized as doctor shoppers in Indiana were for the primary diagnosis of pain. This includes a significant number for unspecified abdominal pain (Table 2), which is also consistent with national data [5].

There are several limitations to our study. First, there a several alternative definitions of doctor shopping that have been used in the literature that significantly differ from ours including defining an individual who used six or more prescribers in a calendar year [11]. It is also possible that patients identified as “doctor shoppers” in our study instead had multiple prescribers due to poor primary care access or required visits to multiple specialists [37]. In addition, the use of Medicaid claims limited our analysis to prescribed opiates and does not consider the entire population of Indiana. Finally, the unintended consequences of policies that reduce prescribing are a concern. We did not consider a possible concurrent shift to illicitly obtained opiates for this group after they were no longer receiving prescription opioids from multiple prescribers. However, although qualitative studies have found that people move to using illicit opioids when they are no longer able to acquire prescription opioids, the occourance in pregnant women has not been quantified. Futher, NIDA reports data from 2011 showing only 4–6% of prescription opioid users moving to heroin [38].

## Conclusion

This study showed that opioid doctor shopping among Medicaid-enrolled pregnant women in Indiana was on the rise a decade after the implementation of the PDMP program (INSPECT) in 2004 and continued following the introduction of the Opioid Prescribing Requirements from the Medical Licensing Board in 2013. However, doctor shopping among this group decreased after passage of Public Law 194, which required INSPECT review prior to the prescribing of any opioids for any duration for all medical practitioners and additionally included potential medical negligence penalties for non-compliance. This shows the importance that legislation addresses single prescriptions and not just long-term opioid prescribing.

## Data Availability

Data used in the analysis as well as all programs used for the analysis may be obtained by contacting the contacting the corresponding author on reasonable request.

## Abbreviations

ICD: International Classification of Diseases
ITS: Interrupted Time Series
INSPECT: Indiana’s Prescription Drug Monitoring Program
MED: Morphine Equivalent Dose
NAS: Neonatal Abstinence Syndrome
SE: Standard Error
